# Analysis of a unique *Clostridium botulinum* strain from the Southern hemisphere producing a novel type E botulinum neurotoxin subtype

**DOI:** 10.1186/1471-2180-12-245

**Published:** 2012-10-31

**Authors:** Brian H Raphael, Matthew Lautenschlager, Suzanne R Kalb, Laura I T de Jong, Michael Frace, Carolina Lúquez, John R Barr, Rafael A Fernández, Susan E Maslanka

**Affiliations:** 1 Centers for Disease Control and Prevention, 1600 Clifton Road, MS G-29, Atlanta, GA, 30329, USA; 2 Area Microbiología, Universidad Nacional de Cuyo, Mendoza, Argentina

**Keywords:** Botulism, Mass spectrometry, Genomics, Whole genome sequencing

## Abstract

**Background:**

*Clostridium botulinum* strains that produce botulinum neurotoxin type E (BoNT/E) are most commonly isolated from botulism cases, marine environments, and animals in regions of high latitude in the Northern hemisphere. A strain of *C. botulinum* type E (CDC66177) was isolated from soil in Chubut, Argentina. Previous studies showed that the amino acid sequences of BoNT/E produced by various strains differ by < 6% and that the type E neurotoxin gene cluster inserts into the *rarA* operon.

**Results:**

Genetic and mass spectral analysis demonstrated that the BoNT/E produced by CDC66177 is a novel toxin subtype (E9). Toxin gene sequencing indicated that BoNT/E9 differed by nearly 11% at the amino acid level compared to BoNT/E1. Mass spectrometric analysis of BoNT/E9 revealed that its endopeptidase substrate cleavage site was identical to other BoNT/E subtypes. Further analysis of this strain demonstrated that its 16S rRNA sequence clustered with other Group II *C. botulinum* (producing BoNT types B, E, and F) strains. Genomic DNA isolated from strain CDC66177 hybridized with fewer probes using a Group II *C. botulinum* subtyping microarray compared to other type E strains examined. Whole genome shotgun sequencing of strain CDC66177 revealed that while the toxin gene cluster inserted into the *rarA* operon similar to other type E strains, its overall genome content shared greater similarity with a Group II *C. botulinum* type B strain (17B).

**Conclusions:**

These results expand our understanding of the global distribution of *C. botulinum* type E strains and suggest that the type E toxin gene cluster may be able to insert into *C. botulinum* strains with a more diverse genetic background than previously recognized.

## Background

There are 7 serotypes (types A-G) of botulinum neurotoxins (BoNT) and types A, B, E or F are the most frequent causes of botulism in humans. Strains of *Clostridium botulinum* producing BoNT/E share similar metabolic characteristics including the inability to digest proteins such as gelatin, casein, or meat. These non-proteolytic strains are psychrophilic with the ability to grow at refrigeration temperatures [1]. In rare cases, strains of *Clostridium butyricum* have been shown to produce BoNT/E [2].

*Clostridium botulinum* type E strains can be isolated from various marine environments and cases of botulism due to BoNT/E typically occur in Canada, Alaska, Northern Europe, and Japan [3]. A total of 56 cases of type E botulism were reported to the Centers for Disease Control and Prevention between 2001–2010 and 87.5% of these cases occurred in Alaska (http://www.cdc.gov/nationalsurveillance/botulism_surveillance.html). Type E botulism has also occurred in the lower 48 states including various outbreaks associated with smoked fish from the Great Lakes [4,5]. A recent outbreak of botulism in birds and fish in the Great Lakes region was attributed to genetically distinct strains of *C. botulinum* type E and the organism was also found in lake sediment [6]. A case of infant botulism occurred in Illinois in 2007 although the source of spores in this case could not be determined [7].

Genetic analysis of 16S rRNA sequences from various *C. botulinum* strains reveals the presence of distinct phylogenetic groups (I-IV) [8,9] which correspond to previously recognized metabolic differences. All Group II strains are non-proteolytic and include type E strains and some type B and type F strains. Nucleotide sequencing of various toxin genes has demonstrated the presence of amino acid variation within genes encoding a single toxin serotype and these variants are identified as toxin subtypes [9,10]. Among type E strains, a total of 8 such subtypes (E1-E8) have been identified [11]. These subtypes differ at the amino acid level by up to 6%.

The genes encoding BoNT/A-G are found in toxin gene clusters that also encode several nontoxic proteins and regulatory proteins. The gene encoding BoNT/E is found within a toxin gene cluster that includes *ntnh* (nontoxic nonhemagglutinin), *p47*, and *orfX1-3*[12,13]. Hill et al. [13] demonstrated that the *bont/E* toxin gene cluster inserted into the *rarA* operon. The transposon-associated gene, *rarA*, likely plays a role in this insertion event in which the gene is split into small and large fragments that flank the toxin gene cluster [13]. Remarkably, an intact *rarA* gene is also located within the toxin gene cluster and the nucleotide sequences of the intact and split genes were shown to differ by phylogenetic analysis. Moreover, the split *rarA* gene fragments can be pasted together to form a gene with a nucleotide sequence with similarity to the gene found in the Group II *C. botulinum* type B strain 17B. In another study, the intact and split *rarA* genes were detected across a panel of 41 type E strains [11].

In this study, we characterized a previously unreported *C. botulinum* type E strain isolated in 1995 from soil in Chubut, Argentina. This represents the first report of a type E strain (CDC66177) originating from the Southern hemisphere. We further show evidence that this strain produces a unique type E toxin subtype and that the genetic background of this strain is highly divergent compared to other type E strains.

## Results and discussion

### Phylogenetic analysis of *bont/E* in *C. botulinum* strains

**Figure 1:**
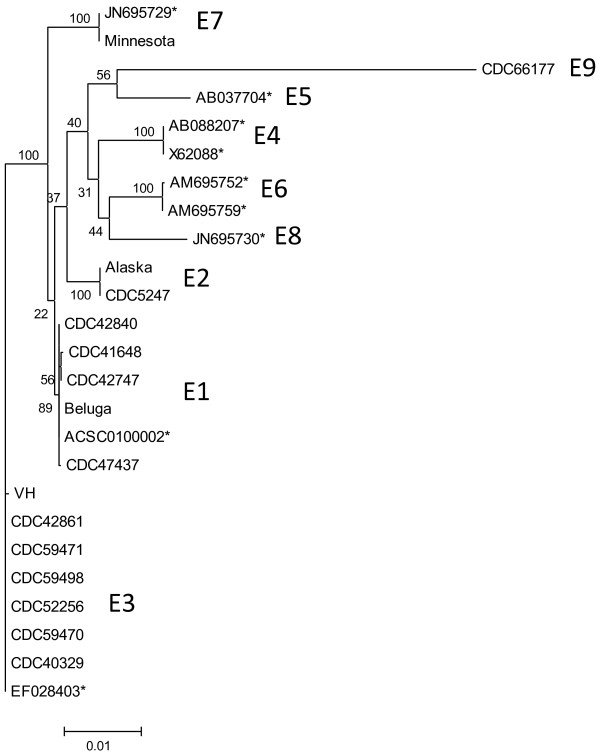
**Dendrogram of *bont/E *nucleotide sequences.** Shown is a neighbor-joining tree of *bont/E* nucleotide sequences with bootstrap values (based on 100 replications) and genetic distance (bar) shown. BoNT/E subtypes (E1-E9) encoded by clusters of genes are also shown. Accession numbers for *bont/E* genes not sequenced in this study are indicated with an asterisk.

The nucleotide sequence of the entire *bont/E* gene was determined for each of the 16 *C. botulinum* type E strains examined in this study. Previous studies have identified several *bont/E* subtypes [9-12]. Nucleotide sequences of *bont/E* determined in this study were compared along with representatives of other reported *bont/E* subtypes (Figure 1). It is important to note that in some cases strain names used in previous reports may not refer to identical strains examined in this study with a similar name. For instance, the CDC reference strain labeled “Alaska” harbored a gene encoding a subtype E2 toxin and is unlikely to be related to the genome-sequenced strain Alaska E43 (Genbank accession number: NC_010723) which encodes a subtype E3 toxin. Another strain labeled “Minnesota” was distinguished from a strain with the same name reported by Macdonald et al. [11]. The CDC Minnesota strain harbored an E7 subtype-encoding gene while the strain examined by Macdonald et al. [11] encoded an E3 subtype toxin.

**Figure 2:**
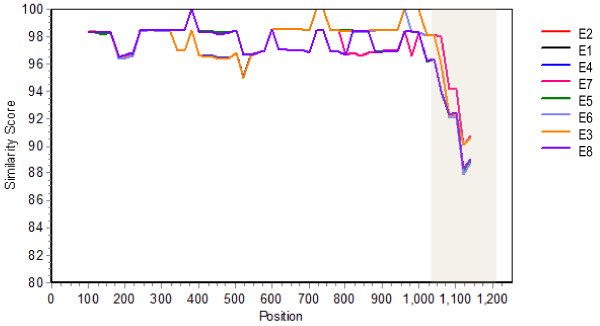
**Comparative analysis of representative BoNT/E subtypes.** Shown is a similarity plot comparing representative BoNT/E subtype amino acid sequences to BoNT/E9 (from strain CDC66177). The most divergent region of the amino acid sequence is shaded. Sequences from representative strains examined in this study or accession numbers retrieved from Genbank are compared in the plot as follows: E1, Beluga; E2, Alaska; E3, CDC40329; E4, AB088207 E5, AB037704; E6, AM695752; E7, Minnesota; E8, JN695730.

Strain CDC66177 harbored a significantly divergent *bont/E* gene which formed a unique clade when compared to other *bont/E* genes. Comparison of the translated amino acid sequence of this gene with the gene encoding BoNT/E1 in strain Beluga indicated that the sequences differed by ~11%. Since previous comparisons of BoNT/E subtypes resulted in differences of up to 6% amino acid sequence variation, the BoNT/E produced by strain CDC66177 can be considered a unique subtype (E9) [10,11]. Comparison of the amino acid sequence of BoNT/E9 with representatives of BoNT/E subtypes E1-E8 demonstrated that the most divergent region of the toxin was located in the last ~200 residues (Figure 2) which corresponds to the C-terminal part of the heavy chain (Hc-C) that is involved with binding to neuronal cells [14]. BLAST analysis of this region indicated < 75% amino acid sequence identity with other BoNT/E sequences.

BLAST analysis of the 16S rRNA nucleotide sequence from strain CDC66177 shared > 99.8% identity with strains Alaska E43 and 17B indicating that the strain clusters with other Group II *C. botulinum* strains [9].

### Mass spectrometric analysis of BoNT/E produced by strain CDC66177

**Figure 3:**
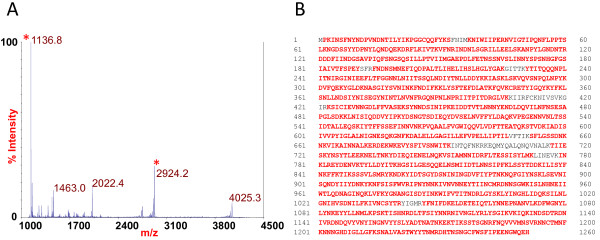
**Mass spectral analysis of BoNT/E9.** Panel **A** shows the products of endopeptidase cleavage of a type E specific peptide substrate detected by mass spectrometry. Peaks indicating the cleavage of the substrate by the toxin are marked with asterisks. Panel **B** illustrates the sequence coverage of BoNT/E9 amino acid sequence determination (in red font) of tryptic digestion products using mass spectrometry.

Since the BoNT/E produced by strain CDC66177 appeared to be a previously unreported toxin subtype, the enzymatic light chain activity of the toxin was assessed in culture supernatants generated from the strain. The light chain of BoNT/E cleaves the synaptosomal-associated protein, SNAP-25, and the Endopep-MS method was used to measure this activity upon a specific peptide substrate mimic of SNAP-25 (IIGNLRHMALDMGNEIDTQNRQIDRIMEKADSNKT). Endopep-MS analysis revealed that the toxin cleaved the peptide substrate for BoNT/E in the expected location, resulting in products with peaks at *m/z* 1136.8 and 2924.2 [15] (Figure 3A).

BoNT/E9 extracted from culture supernatants of strain CDC66177 was subjected to tryptic digestion and the products were analyzed by mass spectrometry to confirm that the toxin's amino acid sequence was indeed unique based on the predicted translation of the DNA sequence. The amino acid sequence of BoNT/E9 was determined with 94.5% coverage (Figure 3B).

### DNA microarray analysis of strain CDC66177

A Group II *C. botulinum* subtyping DNA microarray [16] was used to evaluate gene content in a panel of 21 Group II strains from the CDC culture collection. Briefly, this array featured 495 probes targeting ~15% of the annotated genes in the *C. botulinum* type E strain Alaska E43 and 5 additional probes targeting genes present on the *bont/B*-encoding plasmid (pCLL) in *C. botulinum* type B strain 17B. Genomic DNA isolated from 15 type E strains (not including CDC66177) hybridized with 90.5% of the probes on this array while DNA isolated from type B strains (N=4) and type F strains (N=2) hybridized with 71.9% and 71.0% of the probes, respectively. Genomic DNA from strain CDC66177 hybridized with 66.8% of the probes present on the array.

**Figure 4:**
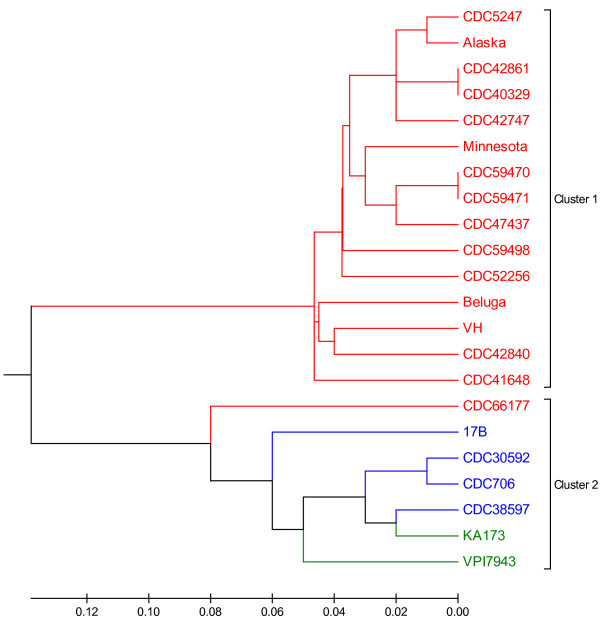
**Microarray analysis of Group II *C. botulinum *strains.** Microarray hybridization profiles of Group II type B, E, and F strains were compared with a UPGMA dendrogram. Type E strains are shown in red, type B strains are shown in blue, and type F strains are shown in green. Cluster 1 consists entirely of type E strains, however, strain CDC66177 groups with Cluster 2.

Comparison of the profile of present or absent genes demonstrated the presence of two clusters of strains (Figure 4). Cluster 1 consisted entirely of type E strains. Interestingly, strain CDC66177 grouped with cluster 2 which included the Group II type B and type F strains examined in this study.

### Southern hybridization of the split *rarA* gene in strain CDC66177

**Figure 5:**
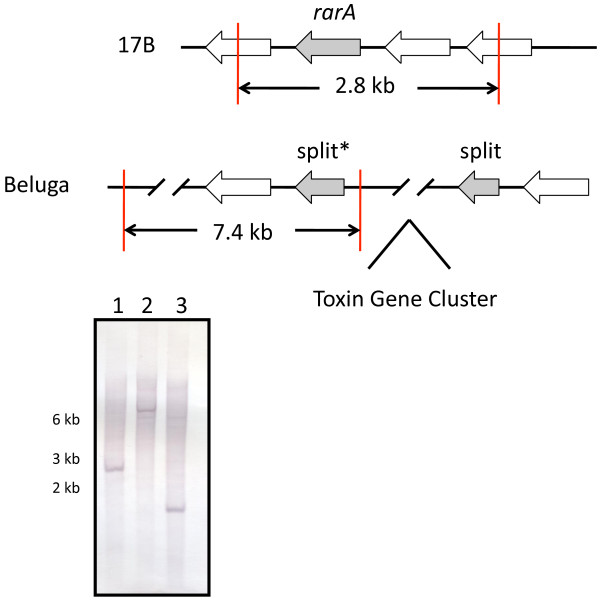
**Southern hybridization of the *rarA *operon.** Schematic representations of the regions surrounding the *rarA* operon are shown. The intact *rarA* gene in strain 17B or the split *rarA* fragments in strain Beluga are shaded. The probe used in the accompanying Southern blot (lane 1, 17B; lane 2, Beluga; and lane 3, CDC66177) targeted either the intact *rarA* gene in strain 17B or the larger *rarA* fragment (indicated by an asterisk) in strain Beluga. *XbaI* restriction sites are indicated by a red line and expected fragment sizes are shown.

In order to determine if the toxin gene cluster in CDC66177 inserted into the *rarA* operon as described for other type E strains [11,13], we performed Southern hybridization using a probe that binds to the larger split *rarA* gene fragment in type E strains or the intact *rarA* gene in the type B strain 17B. Genomic DNA isolated from CDC66177, Beluga, and 17B was digested with *XbaI* and hybridized with the probe. The presence of *XbaI* sites flanking the intact *rarA* gene in strain 17B generated a ~2.8 kb fragment that hybridized the *rarA* probe shown in Figure 5. A ~7.4 kb fragment hybridized with the *rarA* probe in DNA isolated from strain Beluga. These results were expected based on analysis of the *C. botulinum* type E strain Beluga genome sequence (Genbank accession number: ACSC01000002) which demonstrated the presence of separate *XbaI* sites flanking the larger split *rarA* than found at the corresponding intact *rarA* gene in strain 17B (Genbank accession number: NC_010674). The *rarA* probe hybridized a similar size fragment in several other type E strains examined (data not shown). Unexpectedly, a ~1.7 kb band was hybridized by the probe using DNA isolated from strain CDC66177 suggesting the possibility that the regions flanking the toxin gene insertion in this strain were not similar to those of other type E strains.

### Whole genome shotgun sequencing of strain CDC66177

**Figure 6:**
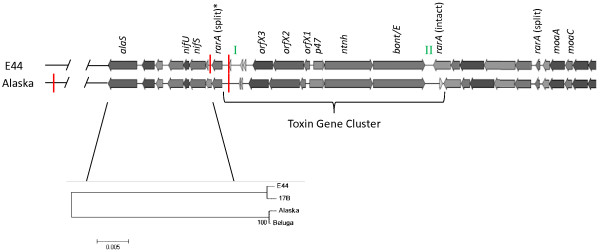
**Organization of the toxin gene cluster and surrounding regions in CDC66177.** The arrangement of genes in the toxin gene cluster and surrounding regions of strain CDC66177 is compared to that of Alaska E43. The toxin gene cluster of strain CDC66177 is located within the *rarA* operon similar to the arrangement in strain Alaska E43. Regions I and II (indicated by green font) contain putative insertion sequences and the location of split and intact *rarA* genes are shown. *XbaI* restriction sites (indicated by red lines) flanking the larger split *rarA* gene (indicated by an asterisk) are shown. The nucleotide sequence between *alaS* and the larger split *rarA* gene of the indicated strains was used to generate the neighbor-joining tree shown.

Since the region flanking the *rarA* operon in strain CDC66177 was suspected to be unlike that of other type E strains, whole genome shotgun sequencing of this strain was performed using the PacBio SMRT sequencer. An ~3.85 Mb draft sequence consisting of 120 contigs was assembled (Genbank accession number: ALYJ00000000). Analysis of this sequence revealed that the toxin gene cluster inserted into the *rarA* operon (Figure 6). The nucleotide sequence of the *bont/E* gene extracted from the genome sequence data was identical to that determined previously by Sanger sequencing. The nucleotide sequence of a ~7.9 kb region starting at *alaS* and extending through CLH_1119 (relative to Alaska E43) was similar to that found in strain 17B but differed from the sequences found in strains Alaska E43 and Beluga.

As shown in Figure 6, the regions between *orfX3* and the larger split *rarA* fragment (region I) and between the smaller split *rarA* fragment and *bont/E* (region II) contain insertion sequences that are likely involved with transposon-mediated mobility of the toxin gene cluster [13]. It is notable that regions I and II differ in size and nucleotide sequence between strains Alaska E43 and CDC66177. In order to determine if the nucleotide sequences of these regions are strain-specific, we also performed an alignment of these regions with strain Beluga. Interestingly, region I in strain Beluga differed from both CDC66177 and Alaska E43 while region II was identical to that found in Alaska E43. While the mechanism of toxin gene cluster insertion into the *rarA* operon is unclear, the sequence similarity in region II between strains Beluga and Alaska E43 suggests at least a partial similarity in the origin of the recombination event that results in the insertion of the toxin gene cluster. However, strain CDC66177 lacks similarity to either strain Beluga or Alaska E43 at either region suggesting that the recombination event resulting in the insertion of the toxin gene cluster in strain CDC66177 originated differently compared to strains Beluga or Alaska E43.

Analysis of the genome sequence data explains the unexpected ~1.7 kb band hybridized by the *rarA* probe in strain CDC66177. The presence of an *XbaI* site within the toxin gene cluster of both CDC66177 and Alaska E43 and an additional site downstream of the larger *rarA* fragment in strain CDC66177 yield an ~1.7 kb fragment. Notably the genome sequence of strain 17B also demonstrates the presence of a *XbaI* site downstream of the intact *rar*A gene. Similar to other type E toxin gene clusters, strain CDC66177 contains an intact *rarA* gene that does not hybridize the *rarA* probe used in our studies. BLAST analysis of this gene demonstrated 98% nucleotide similarity with the gene present in Alaska E43.

Since the *bont/E* gene in strain CDC66177 displayed significant divergence compared to other reported *bont/E* genes, we compared the nucleotide sequences of the remaining toxin gene cluster components (*ntnh, p47, orfX1-3*) to those found in Alaska E43 and Beluga (Table 1). While these genes are nearly identical in Alaska E43 and Beluga, the genes in CDC66177 ranged from 88.2-96.9% nucleotide identity compared to those in Alaska E43 and/or Beluga.

**Table 1:** Pairwise alignment of toxin gene cluster components

**Gene**	**% Nucleotide Identity**		
	**Alaska E43/CDC66177**	**Beluga E/CDC66177**	**Alaska E43/Beluga E**
*orfX3*	94.9	94.9	100
*orfX2*	91.1	91.1	99.5
*orfX1*	94.9	94.9	100
*p47*	88.2	88.2	100
*ntnh*	96.8	96.9	99.9
*bont/E*	93.9	94.1	99.3

In order to further investigate the genomic sequence of strain CDC66177, the average nucleotide identity (ANI) of this strain was compared to Alaska E43 and Beluga. Briefly, 1,020 nucleotide fragments of the query genome were compared to the subject genome using BLAST to determine the ANI value [17]. Richter and Rosselló-Móra [17] proposed an ANI of 95-96% as the boundary of considering two genomes as belonging to a single bacterial species. While comparison of the genomes of strains Alaska E43 and Beluga resulted in an ANI > 97%, comparison of strain CDC66177 with Alaska E43 and Beluga resulted in ANI values between 93-94% (Table 2). Interestingly, comparison of strain CDC66177 with 17B displayed > 98% ANI while comparison of either Alaska E43 or Beluga with 17B resulted in ANI values < 94%. Importantly, only the strain 17B chromosomal sequence was used in these calculations. However, ANI calculations were based on the entire CDC66177 genome sequence since it is unknown if any of the contigs represent mobile elements such as plasmids. Notably, all three strains (Alaska E43, Beluga, and CDC66177), share nearly identical 16S rRNA sequences and clearly cluster with Group II *C. botulinum* (data not shown)*.*

**Table 2:** Average nucleotide identity (ANI) of genomic sequences

**Subject Sequence**^**†**^	**Query Sequence**	**% ANI**
Beluga	CDC66177	93.58
Beluga	17B	93.41
Beluga	Alaska E43	97.91*
CDC66177	Beluga	93.50
CDC66177	17B	98.91*
CDC66177	Alaska E43	93.73
17B	Beluga	93.53
17B	CDC66177	98.97*
17B	Alaska E43	93.67
Alaska E43	Beluga	97.78*
Alaska E43	CDC66177	93.63
Alaska E43	17B	93.50

^†^ The following genome sequences were used in the ANI analysis: Beluga, accession number: ACSC00000000 (4.0 Mb); CDC66177, accession number: ALYJ00000000 (3.85 Mb); 17B, accession number: NC_010674.1 (3.85 Mb); Alaska E43, NC_010723.1 (3.66 Mb).

* ANI values ≥ 96% are marked with an asterisk.

**Figure 7:**
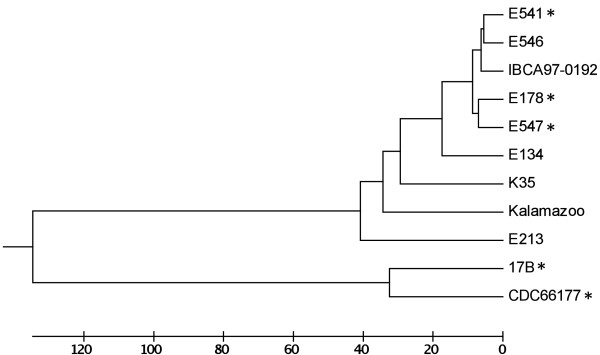
***In silico *analysis of MLST alleles.** Concatemers of MLST alleles for each strain were aligned with CLUSTALW and a UPGMA tree is shown. The scale represents number of differences. Strains isolated from soil and/or sediment sources are indicated with an asterisk. Strain CDC66177 clusters with strain 17B and separately from other type E strains.

Our analysis of the genetic diversity of type E strains using a DNA microarray was limited to those isolated from botulism cases. Therefore, we considered the possibility that strain CDC66177 was genotypically divergent since it was isolated from an environmental source. We performed an *in silico* analysis of multilocus sequence typing (MLST) alleles from selected type E strains (representing isolates from soil and/or sediment, different MLST clades, and different BoNT/E subtypes) reported by Macdonald et al. [11]. These alleles were compared with alleles extracted from the genome sequences of strains 17B and CDC66177. Not surprisingly, strains 17B and CDC66177 formed a separate clade when concatenated MLST alleles were compared to other type E strains (Figure 7).

## Conclusions

In a previous study [18], botulinum toxin-producing clostridia were isolated from 23.5% of soil samples collected in Argentina. The distribution of toxin serotypes reported from the Southern region of Argentina included types A, B, and F. In this study, we characterized a previously unreported *C. botulinum* type E strain (CDC66177) isolated in 1995 from soil collected in Chubut, Argentina. This region is located at a latitude of approximately 43°S which is located as far from the equator as the Great Lakes are located in the Northern hemisphere. While strain CDC66177 was isolated from soil in proximity to the Atlantic Ocean, it is notable that no cases of type E botulism have been reported in Argentina. This is the first known report of the isolation of this strain and extends the known global distribution of *C. botulinum* type E.

While the strain CDC66177 produces a novel BoNT/E subtype, the toxin was shown to cleave a peptide substrate in the same location as other BoNT/E subtypes. It remains to be determined if the toxin produced by this strain varies in its neuronal cell receptor compared to other BoNT/E subtypes. Finally, the presence of *bont/E* in the *rarA* operon of a strain with genetic similarity to strain 17B raises the intriguing possibility of a bivalent non-proteolytic strain expressing BoNT/E encoded by a chromosomally located gene and BoNT/B encoded by a plasmid (such as pCLL found in 17B).

## Methods

### Bacterial strains used in this study

Bacterial strains used in this study are listed in Table 3. Strain CDC66177 was isolated in 1995 from soil collected in Dolavon, Chubut, Argentina (located approximately 58 km from the Atlantic Ocean). The soil sample was originally collected in 1993 in an urbanized area next to a perennial shrub (*Ligustrum sinense*). All *C. botulinum* strains were grown in Trypticase Peptone Glucose Yeast Extract Broth (TPGY) at 35°C under anaerobic conditions.

**Table 3:** Bacterial strains used in this study

**Strain**	***bont*subtype**	**Source**	**Location**	**Year Isolated**	***bont*Accession Number**
Beluga†	E1	Fermented whale	Alaska	1982	GQ244314
CDC41648	E1	Seal flipper	Alaska	1996	JX424539
CDC42747	E1	Stool	Alaska	1997	JX424540
CDC42840	E1	Stool	Alaska	1997	JX424536
CDC47437	E1	Stool	Alaska	1992	JX424545
CDC5247	E2	Fermented seal flipper	Alaska	1984	EF028404
Alaska†	E2	Unknown	Unknown	Unknown	JX424535
CDC52256	E3	Stool	Illinois	2007	GQ294552
CDC59470‡	E3	Stink eggs	Alaska	2004	JX424544
CDC59471‡	E3	Stool	Alaska	2004	JX424542
CDC59498	E3	Stink head	Alaska	2004	JX424543
CDC42861	E3	Seal	Alaska	1997	JX424541
CDC40329	E3	Fish	Alaska	1995	JX424538
VH	E3	Unknown	Unknown	Unknown	GQ247737
Minnesota†	E7	Unknown	Unknown	Unknown	JX424537
CDC66177	E9	Soil	Argentina	1995	JX424534
CDC38597	B4	Blood sausage	Iceland	1983	JX437193
17B†	B4	Marine sediment	Pacific coast, US	1967	EF051570
CDC706	B4	Fermented salmon brine	Alaska	1977	JX437192
CDC30592	B4	Gastric fluid	Alaska	1985	JX437194
KA-173 (610B)	F6	Salmon	Columbia River, US	~1966	GU213230
VPI7943	F6	Venison jerky	California	1966	GU213228

† Strain provided by J. Ferreira (FDA, Atlanta, GA).

‡ Strains are associated with same botulism event.

### DNA extraction, genetic analysis, and DNA microarray

Genomic DNA used in Sanger sequencing and DNA microarrays was extracted using the PureLink Genomic DNA kit (Life Technologies, Grand Island, NY). Neurotoxin and 16S rRNA gene sequences were determined using previously reported primers that amplified overlapping regions [9,19]. Phylogenetic analysis was performed using CLUSTALX and the resulting phylogenetic tree was rendered using MEGA 5.05 [20]. Comparative analysis among representative BoNT/E subtypes was performed using SimPlot (http://sray.med.som.jhmi.edu/SCRoftware/simplot/) with a 200 amino acid window.

The Group II *C. botulinum* subtyping microarray was designed as described elsewhere [16]. Briefly, the microarray featured 495 probes representing genes distributed throughout the *C. botulinum* Alaska E43 genome sequence and 5 additional probes specific for pCLL which encodes the toxin gene cluster in strain 17B. Microarray spotting was performed by ArrayIt (Sunnyvale, CA) or onsite using an Omnigrid Micro microarrayer (Digilab, Holliston, MA). Genomic DNA was labeled with Cy5 random primers and hybridized to the array as previously described [21]. The log of the ratio of the mean fluorescence signal at 635 nm for triplicate probes compared to background fluorescence (locations spotted with buffer alone) was calculated. Log ratios ≥ 1.0 were considered positive and those < 0.5 were considered negative. Log ratios between 0.5 and < 1.0 were considered intermediate likely due to nucleotide sequence variation [21]. Hybridization profiles were converted to binary data by assigning 1 to positive probes and 0 to negative and intermediate probes. Profiles were compared using a UPGMA dendrogram generated with DendroUPGMA (http://genomes.urv.cat/UPGMA/) and selecting the Jaccard coefficient. Microarray data were deposited in the Gene Expression Omnibus with series accession number GSE40271.

### Southern hybridization

Genomic DNA was digested with *XbaI* for 1 h and run on a 1% TBE agarose gel. Alkaline transfer was performed using the TurboBlotter system (Whatman, Kent, ME). An 874 bp probe corresponding to the large *rarA* fragment was generated by PCR amplification with primers RarA-F and RarA-R (RarA-F, 5^′^-GCAAGCACAACTGAAAATCCT-3^′^; RarA-R, 5^′^-CTCGGCTTTTGTXCAATTCATTAG-3^′^) and labeled with the DIG DNA Labeling and Detection kit (Roche, Indianapolis, IN). Hybridization was carried out at 42°C in standard hybridization buffer (5X SSC, 0.1% N-laurylsarcosine, 0.02% SDS, 1% Blocking buffer (from DIG DNA Labeling and Detection kit).

### Mass spectrometric analysis

Botulinum neurotoxin in culture supernatant CDC66177 was extracted and tested for light chain protease activity in a manner similar to that previously described [15], with the exception that 200 μL of culture supernatant was used for this study. Briefly, the neurotoxin was extracted from the culture supernatant using protein G beads coated with antibodies to BoNT/E. Following washing, the beads were then incubated for 4 h at 37°C with a peptide substrate known to be cleaved by BoNT/E in the presence of a reaction buffer. The reaction supernatant was then analyzed by MALDI-TOF mass spectrometry as described previously to determine the location of cleavage of the peptide substrate.

The reaction supernatant was then completely removed from the beads, and the toxin on the beads was digested and analyzed by LC-MS/MS essentially as described previously [22], with the exception that an Orbitrap Elite was used in place of the fourier transform magnetic trap. Briefly, the beads with toxin attached were digested with trypsin and then chymotrypsin. The resultant peptide mixtures were separated by nano-LC and mass analyzed on an Orbitrap Elite, generating MS/MS of the peptides. The MS/MS data were then searched against a database indexed for only *Clostridium* spp. for protein identification.

### Whole genome sequencing and analysis

Genomic DNA was isolated from strain CDC66177 using the MasterPure kit (Epicenter, Madison, WI) with modifications previously described [23]. This DNA was further purified using a Genomic-tip 100/G column (Qiagen, Valencia, CA). One microgram of genomic DNA was sheared using a Covaris S2 ultrasonicator system to a mean size of 1 Kb. The sheared DNA was used to construct a SMRTbell sequencing library (Pacific Biosciences) according to manufacturer’s instructions. The SMRTbell library was then bound into SMRTbell-DNA polymerase complexes and loaded into zero-mode waveguides (ZMW) on 4 SMRTcells and sequenced using Pacific Biosciences C2 chemistry. This relatively small insert sized library was utilized to promote production of circular concensus reads (CCS) which retain higher accuracy base calls than the longer continuous length reads (CLR). Eight 45 min movies were recorded and processed, yielding ~305 K reads with a mean readlength of 2.9 Kbases and total of 889 Mbases of sequence. CCS reads (140 K reads) were then used to error correct the longer (165 K reads) CLR reads [24] utilizing the Pacific Biosciences analysis script BLASR and then the combined CCS/corrected CLR fastq format reads were imported into CLC Genomics workbench. Sequence reads were then trimmed of any remaining Pacific Biosciences hairpin adaptor sequences and quality trimmed to a base Q value of 20. The filtered reads were then assembled *de novo* using the CLC denovo assembler. The 188,898 input reads provided a draft assembly of a 3.85 Mb genome comprised of 119 contigs with an N50 value of 87,742 bases with an average coverage of 28X.

Annotation of the whole genome sequence was performed using RAST [25]. Pairwise alignments of various genes were made with EMBOSS Needle (http://www.ebi.ac.uk/Tools/psa/emboss_needle/nucleotide.html). ANI values were determined using the computer program JSpecies [17]. MLST loci from selected previously reported type E strains were obtained from Genbank [11]. These MLST loci were used to search for the corresponding alleles in the strain 17B genome sequence and the CDC66177 whole genome sequence using BLAST. Concatemers of the alleles for each strain were generated and a multiple sequence alignment was performed using CLUSTALW because the lengths of some alleles in strains 17B and CDC66177 differed due to insertion and/or deletions.

## Competing interests

The authors declare that they have no competing interests.

## Authors’ contributions

LJ and RF isolated strain CDC66177 and performed microbiological characterization. BR and CL designed the study and BR drafted the manuscript. Mass spectral studies were carried out by SK. Genetic studies were carried out by BR and ML. MF performed whole genome sequencing. SM and JB contributed to data analysis and manuscript review. All authors approved the final manuscript.
